# Mandibular Molar Distalization Using Interradicular Microimplants and Indirect Anchorage: A Case Report

**DOI:** 10.7759/cureus.106758

**Published:** 2026-04-09

**Authors:** Fidele Nabbout, Zouhair Skaf

**Affiliations:** 1 Orthodontics, Faculty of Dental Medicine Lebanese University, Beirut, LBN

**Keywords:** class iii, indirect anchorage, mechanics, microimplant, molar distalization

## Abstract

The nonsurgical management of skeletal Class III malocclusion is often challenging, particularly in adolescent or noncompliant patients. Still, temporary anchorage devices have expanded the limits of dentoalveolar compensation by providing reliable skeletal anchorage for distalization of posterior teeth. This case report describes an 18‑year‑old female with skeletal Class III malocclusion, a hypodivergent facial pattern, an anterior crossbite, and compensated mandibular incisors, who was treated with a nonextraction camouflage protocol using microimplant‑supported indirect anchorage to distalize the mandibular molars. The mandibular dentition was distalized with control of the vertical dimension, and clockwise rotation of the mandibular arch, together with mild incisor intrusion, contributed to anterior bite closure while maintaining the compensated inclination of the lower incisors. In this context, Class III correction with microimplant‑supported indirect anchorage for mandibular molar distalization proved to be an effective nonsurgical camouflage alternative to orthognathic surgery, ensuring adequate anterior anchorage control, favorable vertical facial proportions, and an improved occlusal relationship in a carefully selected borderline case.

## Introduction

The origin of a Class III discrepancy may involve mandibular prognathism, maxillary retrusion, or their coexistence, commonly complemented by compensatory dentoalveolar changes [1], including dentoalveolar expansion, which here refers to transverse widening achieved mainly by tooth movement within the alveolar bone rather than by skeletal sutural separation. In borderline skeletal Class III cases, once active growth is nearly complete, or when patients decline combined orthodontic and orthognathic surgery, orthodontic camouflage is commonly selected as an alternative treatment strategy [2-4]. Conventional camouflage approaches, such as Class III elastics or multiloop edgewise mechanics, can correct the anterior crossbite and improve occlusion. Still, these protocols often contribute to additional proclination of the maxillary incisors and may have undesired sequelae on the vertical facial dimension [1-4].

Over recent decades, temporary anchorage devices have significantly modified anchorage management and expanded the envelope of dentoalveolar compensation [1,5-7]. Microimplants provide skeletal anchorage, enabling predictable distalization or intrusion of teeth with reduced dependence on patient cooperation and fewer adverse effects on the anterior dentition [5]. While maxillary molar distalization with skeletal anchorage is well documented, distal movement of lower molars is more demanding due to bone density, space limitations, and the proximity of important anatomical landmarks such as the mandibular canal [8,9].

Temporary anchorage devices placed in the mandibular buccal cortex enable the construction of indirect anchorage systems that distalize the posterior segment while limiting loss of anterior anchorage. Indirect anchorage refers to a setup in which microimplants stabilize the posterior teeth via the archwire rather than serving as the direct site of force application. When attempting a camouflage treatment for skeletal Class III cases, it is crucial to maintain or slightly improve mandibular incisor inclination and preserve the vertical dimension to achieve a favorable functional and esthetic result [1-4,6]. When carefully designed, microimplant‑assisted mechanics enable the application of a precisely controlled force vector relative to the centers of resistance of the dental segments, permitting distalization of lower molars with simultaneous control of incisor position and facial height; the center of resistance is the point in a tooth or dental segment at which a single force produces translation without rotation [5-7,10-12].

This case report documents the orthodontic management of a borderline skeletal Class III adolescent patient who refused orthognathic surgery and was treated with microimplant‑supported indirect anchorage for mandibular molar distalization to illustrate the biomechanical aspects and clinical implications of this nonsurgical camouflage approach [5,6,11].

## Case presentation

**Figure 1:**
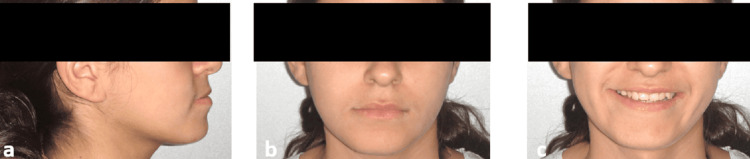
Pre-treatment extraoral photographic documentation (a) Right view, (b) frontal view at rest, (c) frontal smiling view.

An 18‑year‑old female complained of an unesthetic smile due to an anterior crossbite. Extraoral examination showed a slightly concave profile (Figure 1a), balanced vertical facial proportions, and competent lips, indicating a mild Class III tendency in profile (Figure 1a-1c).

**Figure 2:**
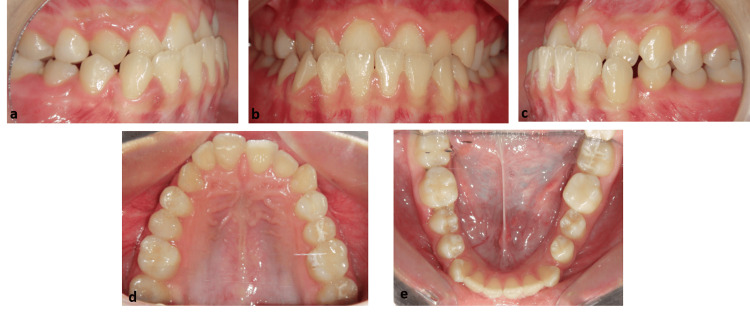
Pre-treatment intraoral photographic documentation (a) Right buccal view, (b) frontal view in maximum intercuspation, (c) left buccal view, (d) maxillary occlusal view, (e) mandibular occlusal view.

Intraoral assessment revealed bilateral full-step Class III molar and canine relationships (Figure 2a, Figure 2c), a crossbite anteriorly (Figure 2b), and mild crowding in the mandibular arch (Figure 2e), while having the mandibular molars positioned relatively mesial to the maxillary arch. The overjet was negative, and the mandibular midline was shifted 3 mm to the right. The occlusal views of the maxillary and mandibular arches reveal well‑aligned dental segments with mild arch‑form asymmetries and slight crowding in the lower anterior region (Figure 2d-2e).

**Figure 3:**
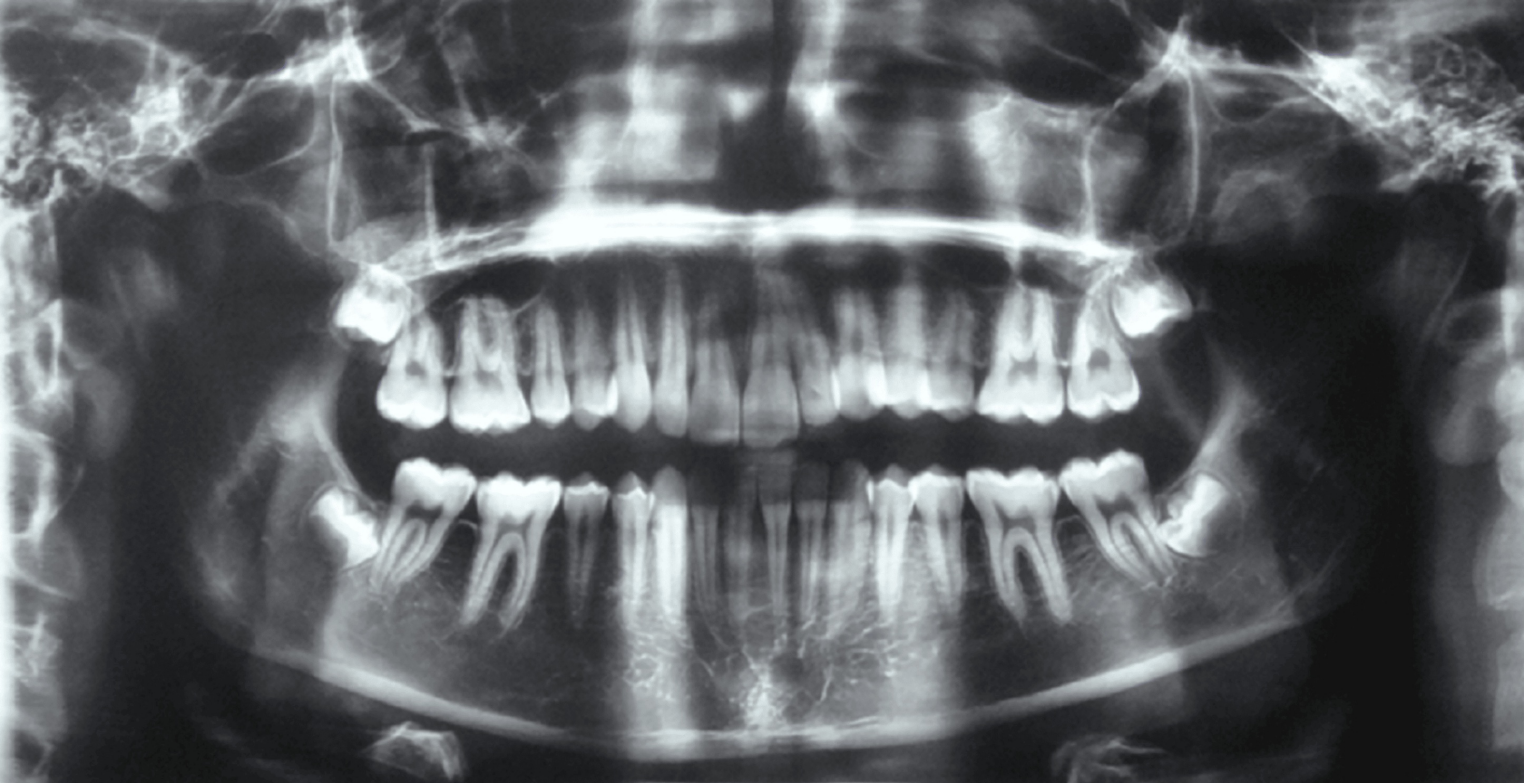
Pre-treatment panoramic radiographic assessment Panoramic X-ray illustrating the baseline status of the dentition and supporting osseous structures.

Panoramic radiography confirmed the presence of all permanent teeth with normal root morphology and no evidence of caries, periodontal disease, or root resorption (Figure 3).

**Figure 4:**
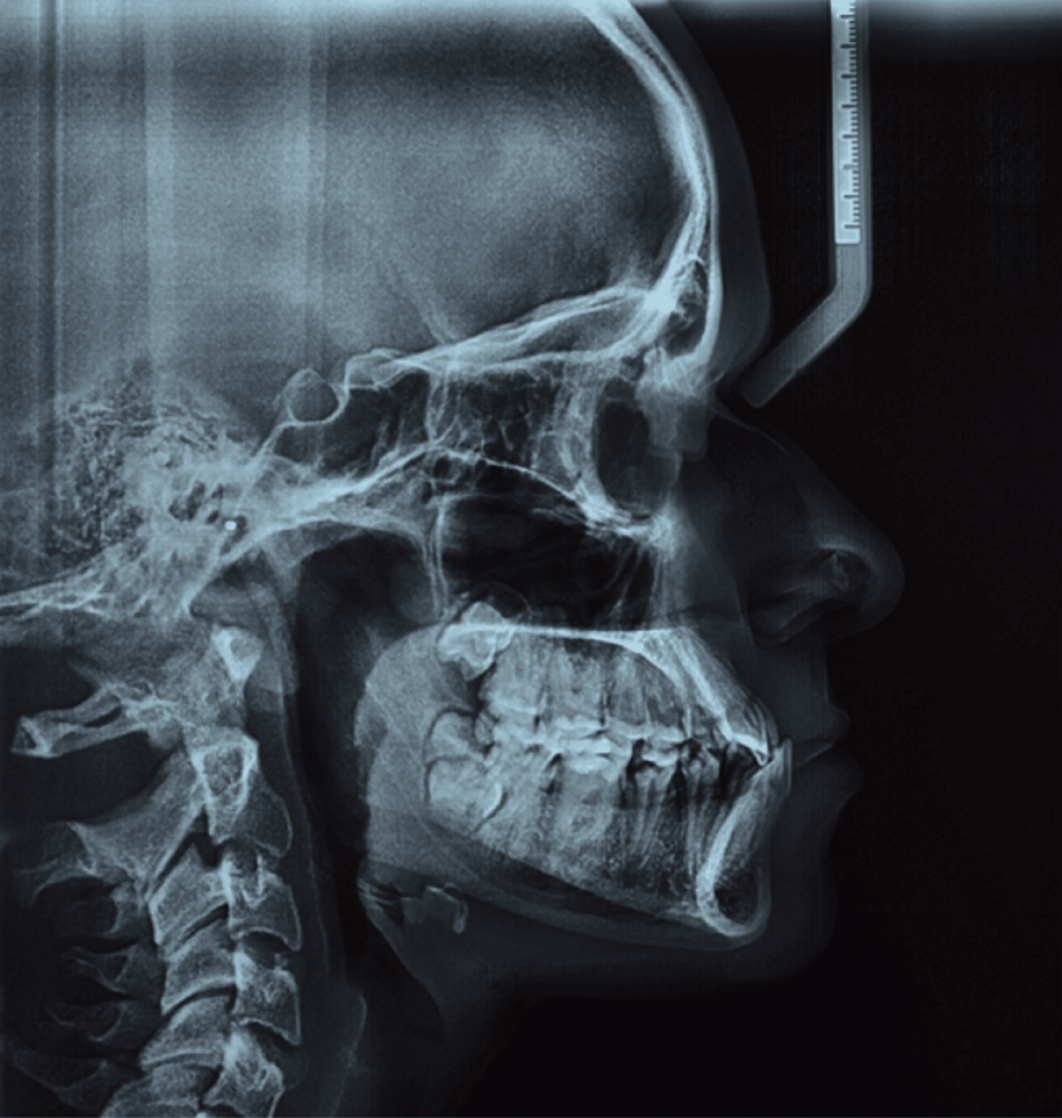
Pre-treatment lateral cephalometric analysis Lateral cephalometric radiograph depicting the initial skeletal pattern and dentoalveolar relationships.

Lateral cephalometric evaluation (Figure 4, Table 1) demonstrated a skeletal Class III aspect characterized by an ANB angle of −3°, an AoBo discrepancy (Wits appraisal) of −7 mm, a low to normal mandibular plane angle, and mild compensatory dentoalveolar variations. The mandibular incisors were proclined relative to the NB line as well as to the A-Pog line. In contrast, the maxillary incisors were positioned within normal limits, indicating a Class III discrepancy mainly related to mandibular prognathism with compensated mandibular incisors [1,8].

**Table 1:** Assessment of cephalometric variables Cephalometric measurements obtained pre- and post-treatment, presented relative to commonly used normative values, including reference soft‑tissue values from the Tweed–Merrifield analysis. SNA: sella–nasion–A point angle, SNB: sella–nasion–B point angle, ANB: A point–nasion–B point angle, FMA: Frankfort–mandibular plane angle, IMPA: incisor–mandibular plane angle, FMIA: Frankfort–mandibular incisor angle, U1-FH: upper incisor to Frankfort horizontal plane angle, U1-L1: upper incisor to lower incisor angle

Variables	Norm	Pre-treatment	Post-treatment
SNA	82°	89°	89°
SNB	80°	92°	91°
ANB	2°	-3°	-2°
Wits appraisal	0mm	-7mm	-5mm
FMA	25°	12°	12°
IMPA	88°	100°	93°
FMIA	67°	68°	75°
U1-FH	110°	117°	118°
U1-L1	135°	116°	120°
Occlusal plane	10°	8°	9°
Z angle	75°	85°	87°
Upper lip	mm	10	8
Total chin	mm	10	12

This borderline skeletal Class III case, with a hypodivergent pattern, bilateral full-step Class III molar and canine relationships, anterior crossbite, and proclined mandibular incisors, was managed nonsurgically with mandibular molar distalization camouflage.

Treatment goals

The treatment objectives were to correct the anterior crossbite and establish a positive overjet and normal overbite, distalize the mandibular buccal segments to achieve bilateral Class I relationships, maintain or slightly enhance the facial profile without increasing the vertical dimension, and control mandibular incisor inclination to maintain or slightly improve their compensated position and avoid additional proclination during camouflage correction [4-6].

Given the borderline skeletal discrepancy and the patient’s refusal of orthognathic surgery, a non‑extraction camouflage approach was adopted. This strategy relied on Class III correction using microimplant‑supported indirect anchorage for mandibular molar distalization. The treatment protocol involved distalizing the mandibular posterior segment with skeletal anchorage to create space for alignment and to allow spontaneous distal drift of the premolars, followed by en masse retraction of the anterior segment while maintaining control of mandibular incisor inclination.

Treatment procedure

**Figure 5:**
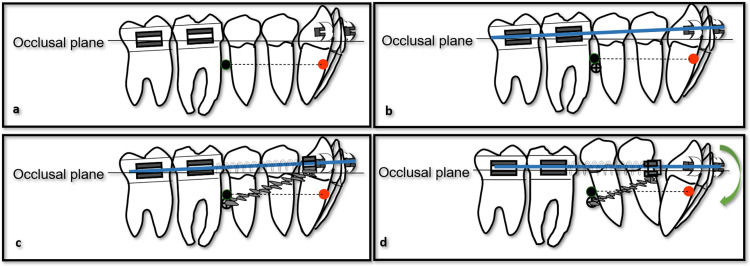
Schematic illustration of the mandibular molar distalization biomechanics with microimplant‑supported indirect anchorage (a) Initial setup with a rigid mandibular posterior segment (premolars bypassed) on a 0.019 × 0.025‑inch stainless‑steel archwire. The circle in black indicates the center of resistance of the mandibular arch, while the circle in red represents that of the anterior sector. (b) Placement of bilateral buccal microimplants mesial to the first molars and connection to sliding hooks distal to the canines, creating an indirect anchorage unit that reinforces the posterior teeth via the archwire. (c) Activation of nickel‑titanium open‑coil springs between the sliding hooks and posterior segment, with forces transmitted through the archwire to produce distalization of the mandibular molars. (d) The resulting combination of nearly translational distal movement of the molars, mild intrusion of the mandibular incisors, and slight clockwise rotation of the mandibular arch contributes to sagittal correction and anterior bite closure with vertical control. Image Credit: Author using PowerPoint (Microsoft Corp., Redmond, WA, USA)

Preadjusted MBT prescription 0.022-inch slot fixed appliances (Mini Uni-Twin, 3M, Monrovia, CA) were bonded on both arches. In the mandibular arch, the first and second premolars were initially left unbonded to facilitate construction of a rigid posterior segment (Figure 5a). Leveling and alignment were accomplished with a sequence of nickel-titanium and stainless steel archwires, progressing up to a 0.018 × 0.025-inch stainless steel archwire in the mandible. The maxillary arch was aligned, then coordinated with standard mechanics to decompensate the occlusion and prepare for interarch coordination.

Following initial leveling, the insertion of a 0.019 × 0.025-inch stainless steel archwire in the mandibular arch while bypassing the premolars created a stiff posterior unit suitable for distalization. Two buccal microimplants 1.4 mm x 8 mm (AbsoAnchor, Dentos, Inc., Daegu, Korea) were engaged at the mucogingival junction mesial to the mandibular first molars (Figure 5b). Heavy steel ligatures were used to connect the microimplants to hooks soldered distal to the canines, creating an indirect anchorage system in which the microimplants reinforced the posterior segment through the archwire. The overall biomechanical setup, including the occlusal plane, the centers of resistance of the whole mandibular arch and the anterior segment, and the line of action of the forces from the microimplants, was designed to ensure proper control of the force vector [7,10-12], while maintaining an acceptable miniscrew failure risk [13].

Open-coils were installed between sliding hooks on the mandibular archwire and the posterior segment and activated with steel ligatures tied to the microimplants to distalize mandibular molars (Figure 5c). The following is a microimplant-supported indirect anchorage configuration that minimized anterior anchorage loss and allowed controlled, near-translation distalization of the first and second molars. The described model resulted in a combination of distal displacement of the mandibular molars, intrusion of the mandibular incisors, and a clockwise rotation of the mandibular arch (Figure 5d), in line with evidence that skeletal anchorage-based treatments in skeletal Class III patients provide greater ANB and Wits improvement, better vertical control, and fewer dentoalveolar side effects than conventional anchorage protocols [14].

**Figure 6:**
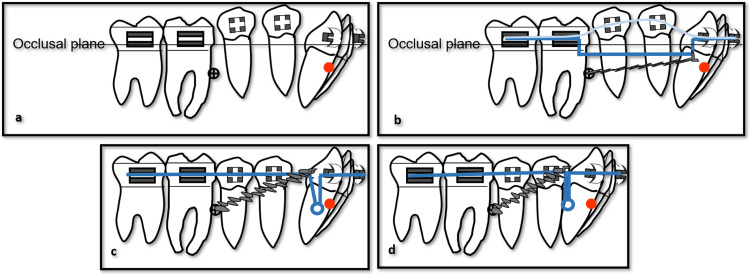
Final-stage biomechanical schematic illustrating mandibular distalization and vertical control (a) Distal placement of microimplants and bonding and leveling of the mandibular premolars. (b) A steel ligature was connected between the microimplant and hooks soldered distal to the canines, together with a 0.016-inch NiTi overlay wire to level the premolars. (c) Following premolar uprighting and posterior space closure, incisor retraction was performed using loop mechanics, with posterior anchorage reinforced by a steel ligature connecting the distal leg of the closing loop to the microimplant. (d) Final configuration after completion of incisor retraction. Image Credit: Author using PowerPoint (Microsoft Corp., Redmond, WA, USA)

As distal space became available, the mandibular premolars showed spontaneous distal drift into these spaces, improving the efficiency of space management [11,15]. Additional steps, such as optional distal repositioning of the microimplants, bonding and leveling of the premolars, the use of a 0.019 × 0.025-inch stainless steel archwire in addition to step-ups, and a 0.016-inch nickel-titanium overlay wire, were applied to upright premolars and close residual posterior spaces (Figure 6a-6b). The anterior closing loop was activated to retract the mandibular incisors en masse while maintaining torque control and consolidating space created by posterior distalization (Figure 6c-6d).

**Figure 7:**
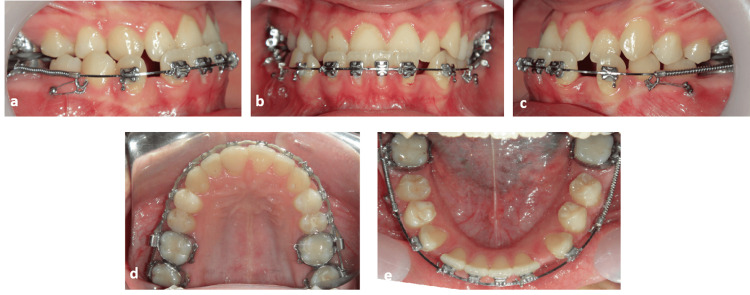
Intraoral photographs obtained through active treatment (a) Right buccal view, (b) frontal view in maximum intercuspation, (c) left buccal view, (d) maxillary occlusal view, (e) mandibular occlusal view.

After completion of distalization and closure of the posterior spaces, the mandibular anterior teeth were retracted en masse, utilizing the same microimplant-supported indirect anchorage concept. Steel ligatures from the microimplants to the posterior segment reinforced posterior anchorage, while retraction forces were applied from hooks in the anterior region [16]. Sequential intraoral photographs documented progressive resolution of the anterior crossbite, improvement in overjet and overbite, and the establishment of a Class I canine relationship (Figure 7a-7c). Finishing, in addition to detailing procedures, was performed with coordinated archwires and light interarch elastics to refine occlusal intercuspation and coordinate midlines (Figure 7d-7e).

**Figure 8:**
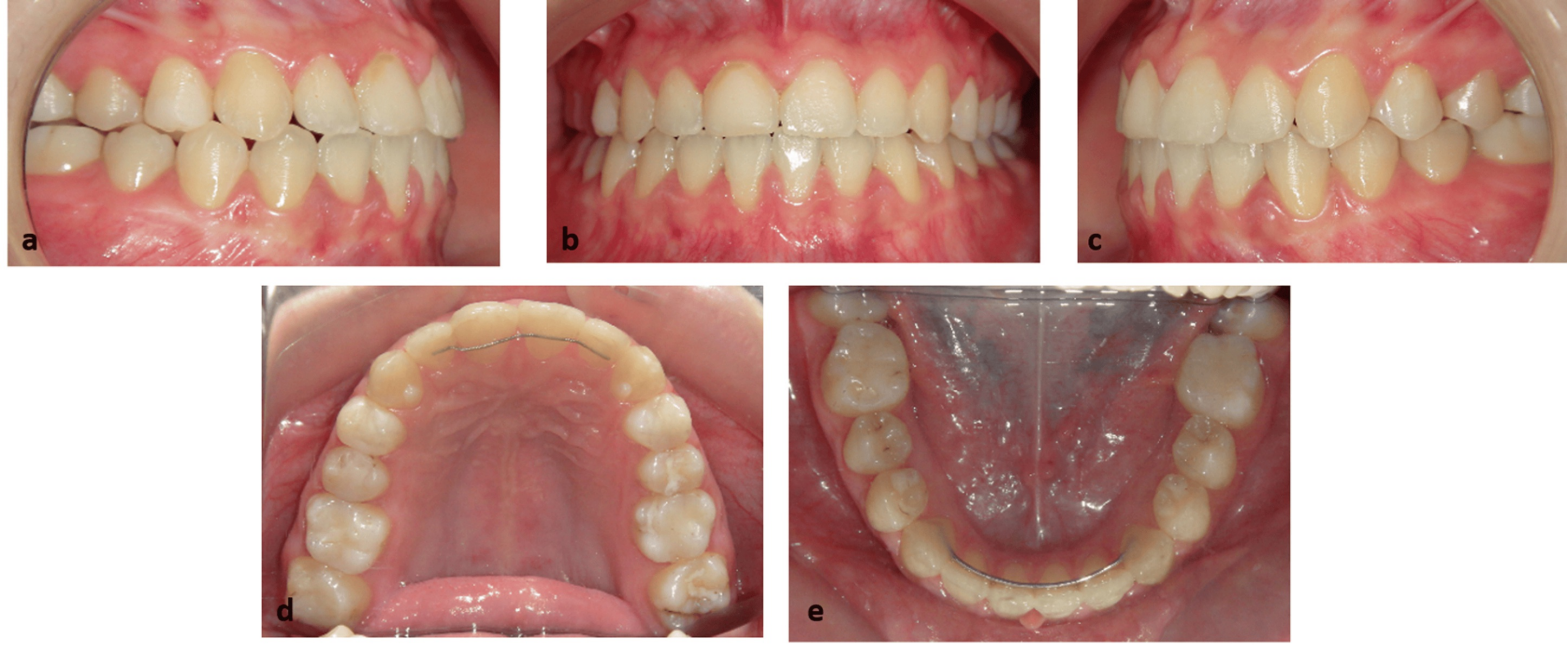
Post-treatment intraoral photographic documentation (a) Right buccal view, (b) frontal view in maximum intercuspation, (c) left buccal view, (d) maxillary occlusal view, (e) mandibular occlusal view.

Fixed appliances were removed once stable Class I relationships and satisfactory functional contacts had been obtained. Treatment was completed after 24 months, and fixed and Essix retainers were delivered on both dental arches (Figure 8d-8e). Afterward, the patient underwent the removal of lower third molars.

Treatment outcomes and evaluation

Post-treatment, a bilateral Class I molar and canine relationship was present, together with normalized overjet and overbite. Final intraoral photographs demonstrated well-aligned maxillary and mandibular arches, appropriate transverse coordination, and good intercuspation with stable Class I occlusion (Figure 8a-8c).

**Figure 9:**
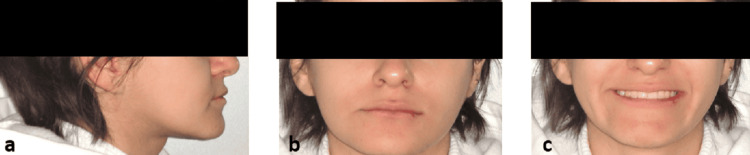
Post-treatment extraoral photographic documentation (a) Right view, (b) frontal view at rest, (c) frontal smiling view.

Post-treatment extraoral photographs showed a more harmonious facial profile, with resolution of the anterior crossbite and a maintained vertical dimension (Figure 9a-9c).

**Figure 10:**
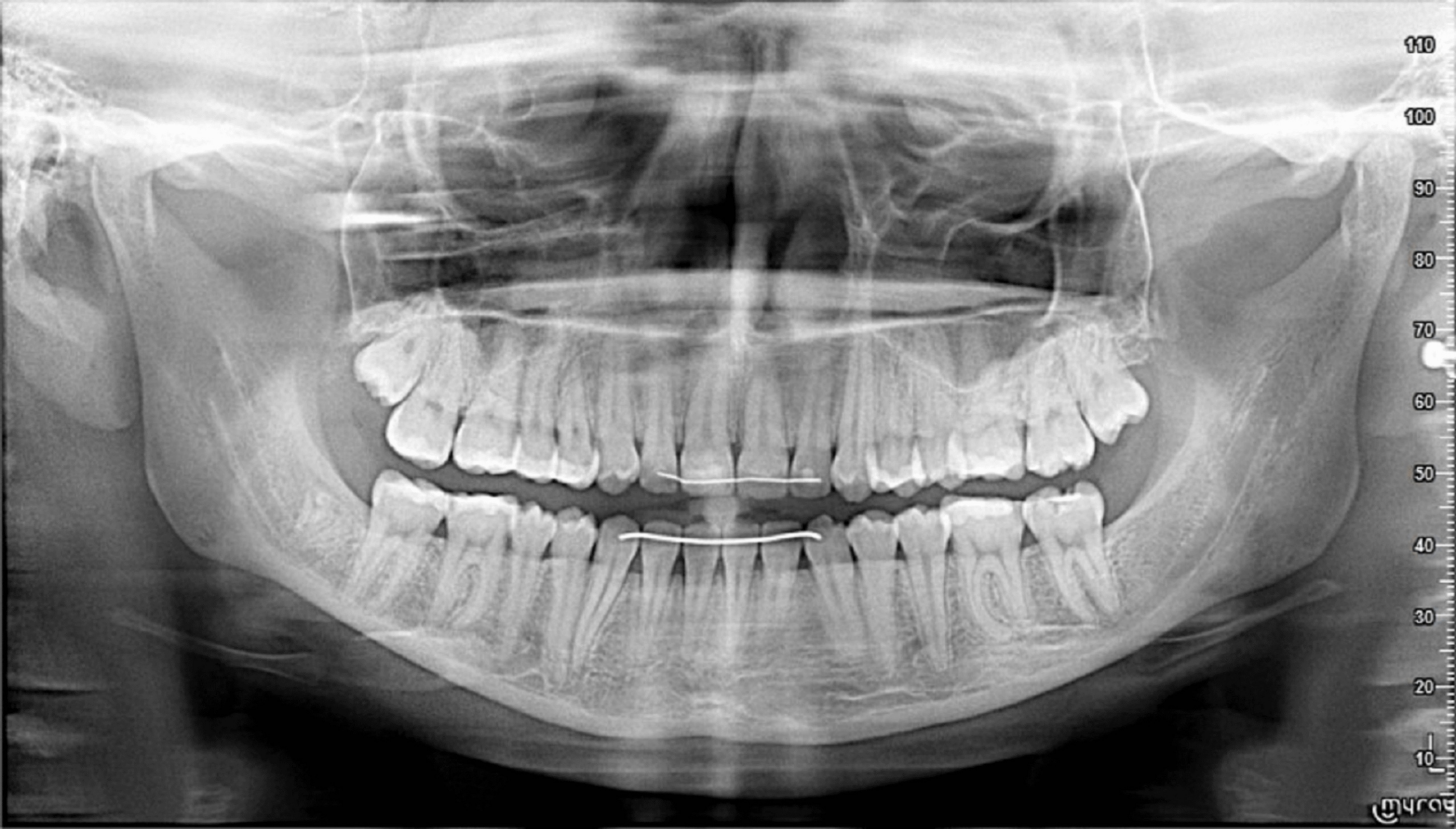
Post-treatment panoramic radiograph Panoramic radiograph taken after completion of orthodontic treatment, showing well‑aligned maxillary and mandibular arches, good root parallelism, and no evident root resorption or other radiographic abnormalities.

The final panoramic radiograph demonstrated good root parallelism, with no significant root resorption or other adverse effects (Figure 10).

**Figure 11:**
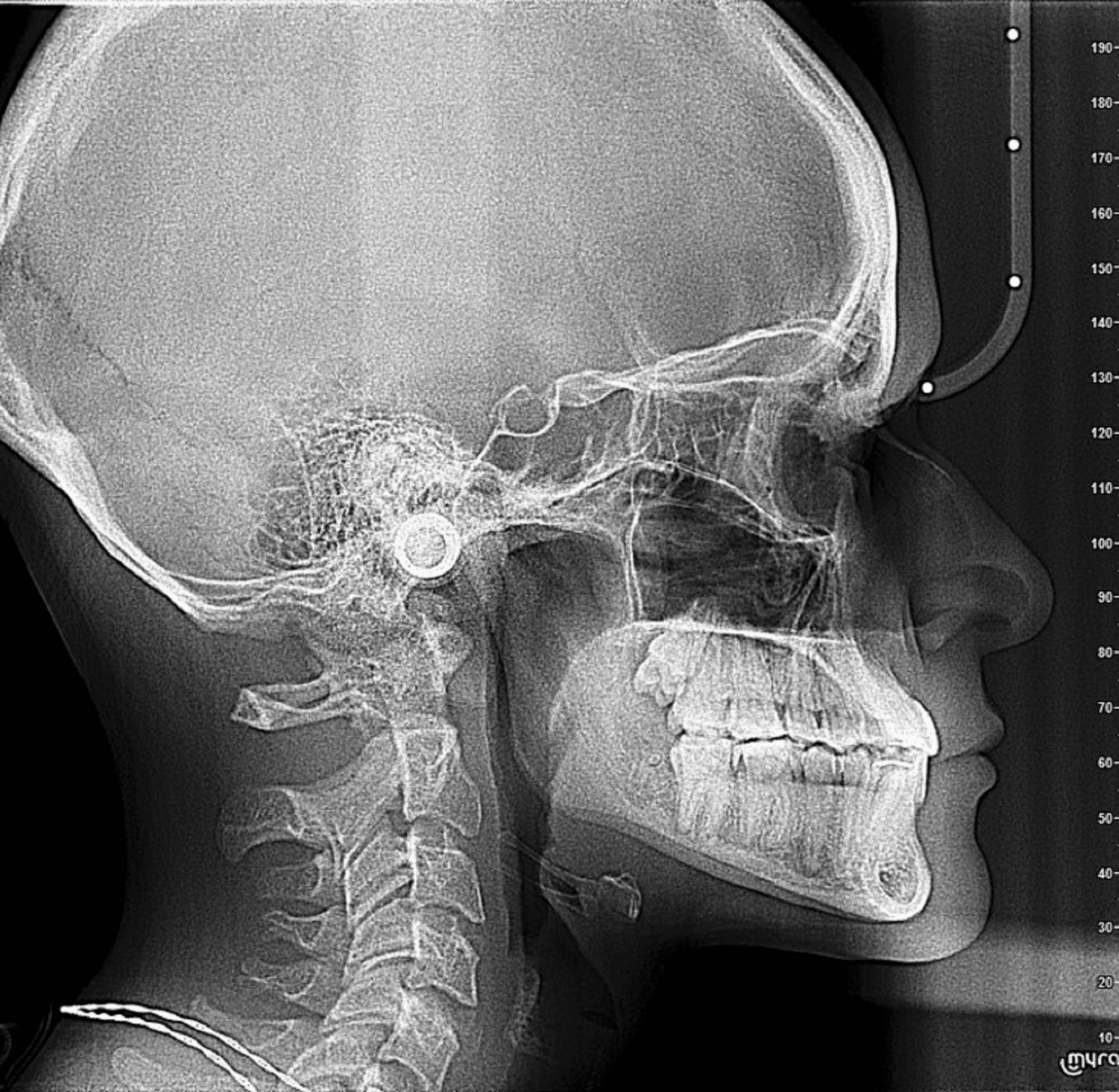
Post-treatment lateral cephalometric radiograph Lateral cephalometric radiograph obtained at the end of treatment, demonstrating a balanced facial profile, corrected incisor relationship, and stable skeletal relationships with minimal change in mandibular plane inclination.

Comparison of pre-treatment and post-treatment lateral cephalometric radiographs and tracings (Figure 11, Table 1) confirmed that the principal changes were dentoalveolar, with distal positioning of the mandibular molars, maintenance or slight improvement of mandibular incisor inclination, and minimal alteration of the mandibular plane angle. The cephalometric summary (Table 1) showed an improvement in the ANB angle from −3° to −2°, a reduction in the AoBo discrepancy, and a more favorable position of the mandibular incisors relative to the NB and A-Pog lines.

## Discussion

This report indicates that Class III correction with microimplant-supported indirect anchorage for mandibular molar distalization can be a useful nonextraction camouflage strategy in mild to moderate skeletal Class III malocclusions and a hypodivergent growth pattern, particularly when orthognathic surgery is refused or not clearly indicated [2-4]. Distalization of mandibular molars is biomechanically challenging owing to the dense mandibular cortical bone, limited distal space, and proximity to vital structures such as the mandibular canal [13-15,17]. The presence of the mandibular third molars did not hinder the distalization process since their buds were deeply positioned [9,15-16]. Distalization appliances originally designed for the maxilla cannot be transferred to the mandible without significant modification of the biomechanics [10-12,18]. The use of skeletal anchorage with microimplants or miniplates has enabled mandibular molar distalization in selected adolescent and adult patients by providing a stable anchorage unit that does not rely heavily on patient cooperation [19,20].

In the present case, the microimplant-reinforced indirect anchorage system generated a controlled force system that combined distalization of the mandibular molars, mild incisor intrusion, and a slight clockwise rotation of the mandibular arch. The following pattern of movement contributed to sagittal correction while limiting deterioration of the vertical dimension and preventing further retroclination of the mandibular incisors, a key factor in the camouflage management of skeletal Class III malocclusion. Clinically, this was manifested by minimal clockwise rotation of the mandibular plane while preserving the vertical dimension. The following type of mechanics, i.e., indirect anchorage, is mainly indicated in Class III cases with normodivergent or hypodivergent facial patterns, by contrast to the Class III cases with normodivergent or hyperdivergent facial patterns, where direct anchorage was used, as demonstrated in our previous paper.

**Figure 12:**
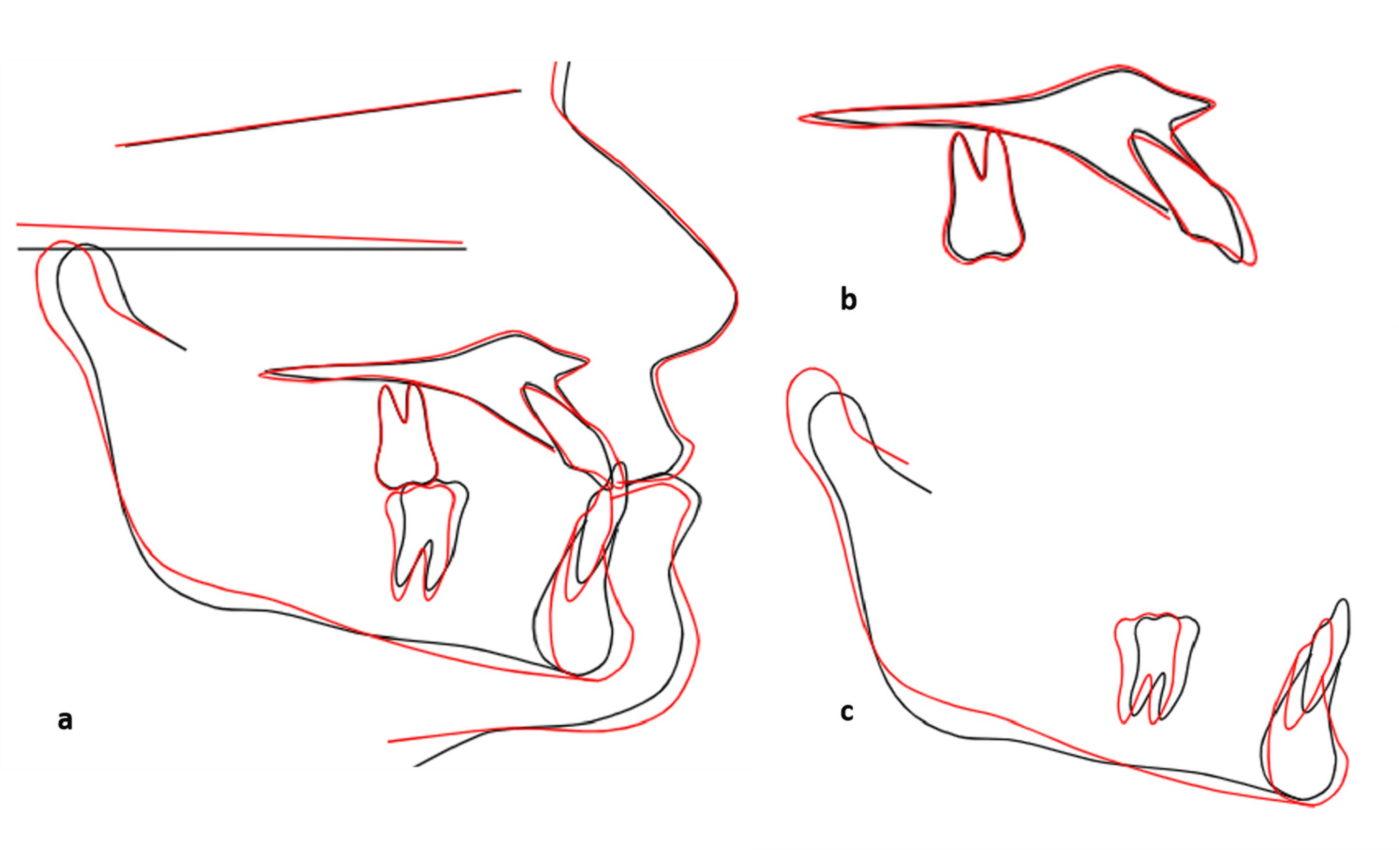
Cephalometric superimposition analysis showing superimposition of pre-treatment (black) and post-treatment (red) tracings (a) Overall superimposition on the anterior cranial base, (b) regional maxillary superimposition highlighting positional changes in incisors and molars, (c) regional mandibular superimposition demonstrating tooth movement within the mandibular arch. Image Credit: Author using tracings obtained manually using conventional cephalometric tracing on acetate

The superimposed tracings demonstrate predominantly dentoalveolar changes, with distal displacement of the mandibular molars, maintenance of mandibular incisor inclination, and minimal alteration of the mandibular plane and overall facial profile (Figure 12a-12c).

The observed spontaneous distal drift of the premolars into the spaces created by molar distalization increased treatment efficiency by reducing the need for direct distalization mechanics on these teeth. Subsequent en masse retraction of the anterior segment, favored by indirect anchorage due to microimplants and posterior teeth, allowed precise control of incisor position and torque (Figure 6c-6d). These outcomes are consistent with the concept of dentoalveolar camouflage in skeletal Class III malocclusion and with previous studies reporting that mandibular molar distalization using skeletal anchorage can be effective when case selection, microimplant positioning, and biomechanical design are carefully planned. Despite these advantages, this technique requires meticulous evaluation of available distal space, root proximity, and mandibular anatomy, as well as accurate placement of microimplants to avoid root contact, soft-tissue irritation [13], or microimplant failure. Long-term follow-up is mandatory to assess the sagittal and vertical stability of mandibular molar distalization. Still, current evidence suggests that skeletal anchorage-based protocols can provide acceptable stability when the indication is appropriate, and mechanics are well controlled [14-16,20]. These qualitative changes appear to fall within the range of dentoalveolar effects previously reported for mandibular molar distalization using skeletal anchorage in Class III camouflage cases.

This report has several limitations that should be acknowledged. As a single‑case description, it does not allow for generalization of the outcomes or for statistical comparison with alternative treatment modalities. The follow‑up period is also limited, so longer‑term skeletal and dentoalveolar stability, particularly of the distalized mandibular molars, cannot be fully assessed. Additionally, case selection and patient‑specific factors may have favored a positive response to camouflage with skeletal anchorage, which introduces an inherent selection bias. Future prospective studies with larger sample sizes and extended follow‑up would be valuable for confirming the reproducibility and stability of this treatment approach.

## Conclusions

Class III correction with microimplant-supported indirect anchorage for mandibular molar distalization appears to be a predictable and efficient nonsurgical camouflage technique for selected skeletal Class III malocclusions. In the case presented, the applied biomechanics allowed controlled distalization of the mandibular molars, spontaneous distal drifting of the premolars, and en masse retraction of the anterior teeth with minimal anterior anchorage loss and satisfactory vertical control. This approach broadens the spectrum of nonextraction camouflage options and may reduce the need for orthognathic surgery when indications, microimplant placement, and force systems are carefully considered.
